# Role of the TRPM4 Channel in Cardiovascular Physiology and Pathophysiology

**DOI:** 10.3390/cells7060062

**Published:** 2018-06-15

**Authors:** Chen Wang, Keiji Naruse, Ken Takahashi

**Affiliations:** Department of Cardiovascular Physiology, Graduate School of Medicine, Dentistry and Pharmaceutical Sciences, Okayama University, Okayama 700-8558, Japan; wangchen11228@gmail.com (C.W.); knaruse@md.okayama-u.ac.jp (K.N.)

**Keywords:** TRPM4 channel, cardiovascular system, physiology, pathophysiology

## Abstract

The transient receptor potential cation channel subfamily M member 4 (TRPM4) channel influences calcium homeostasis during many physiological activities such as insulin secretion, immune response, respiratory reaction, and cerebral vasoconstriction. This calcium-activated, monovalent, selective cation channel also plays a key role in cardiovascular pathophysiology; for example, a mutation in the TRPM4 channel leads to cardiac conduction disease. Recently, it has been suggested that the TRPM4 channel is also involved in the development of cardiac ischemia-reperfusion injury, which causes myocardial infarction. In the present review, we discuss the physiological function of the TRPM4 channel, and assess its role in cardiovascular pathophysiology.

## 1. Introduction

The transient receptor potential (TRP) melastatin-like subfamily member 4 (TRPM4) is a 1214-amino-acid-long transmembrane protein encoded by *TRPM4*, which is located on human chromosome 19 [1,2]. As a member of the TRP family, it participates in mediating the flux of Na^+^ and K^+^ across the plasma membrane into the cytoplasm [3]. In contrast to most of the functionally characterized TRP channels, which are nonselective Ca^2+^-permeable cation channels, TRPM4 and TRPM5 are permeable only to monovalent cations, and not to Ca^2+^ or Mg^2+^. The Ca^2+^ impermeability of the TRPM4 channel plays a role in the accumulation of intracellular Ca^2+^, which leads to the depolarization of the plasma membrane [2,3,4,5]. Although it is impermeable to Ca^2+^, TRPM4 is a Ca^2+^- and voltage-activated channel, and its activation is regulated via a variety of methods. Phosphatidylinositol 4,5-bisphosphate (PIP_2_) is an effective modulator of TRPM4 as the channel can be rapidly desensitized to intracellular Ca^2+^ [6,7]. In addition, adenosine triphosphate (ATP), protein kinase C (PKC)-dependent phosphorylation, and calmodulin (CaM) also play a role in TRPM4 activation [8,9,10,11], which will be discussed later on in the review.

Like other TRP channels, TRPM4 also comprises six transmembrane domains. The NH_2_ and COOH terminal regions of TRPM4 contain binding sites that are related to the channel’s activation [1,10]. TRPM4 is highly expressed in a number of tissues and organs and involved in complicated physiological and pathological mechanisms, especially in calcium-dependent mechanisms, such as insulin secretion, immune response, respiratory reaction, tumor development, and cardiovascular diseases [12,13,14,15]. Cardiovascular risks have a detrimental impact on human health, and some research has implicated TRPM4 in cardiac hypertrophy, myocardial ischemia-reperfusion injury (IRI), and hereditary arrhythmia [16,17,18,19,20,21,22,23,24]. In the present review, we focus on the physiological role of TRPM4, and discuss its involvement in cardiovascular pathophysiology.

## 2. Physiological Characteristics of TRPM4

### 2.1. TRP Overview

TRP cation channels were first reported in *Drosophila* spp. in 1975, and over 50 family members have been characterized to date [1,25]. In general, they can be divided into seven subfamilies in accordance with the amino acid sequence homology: TRPC, TRPV, TRPM, TRPP, TRPML, TRPA, and TRPN. At present, 28 genes of the TRP family have been found to be expressed in mammalians in succession, but only 27 different members are in humans [1,3,26]. The biophysical properties of TRP family members have been described previously according to the reviews of Clapham et al., Watanabe et al., and Zheng et al. [11,15,26,27]. As mentioned earlier, all the TRPs contain six transmembrane domains and are tetramerized to form functional channels. Therefore, the classical structure of multiple cation channels is mimicked, such as voltage-dependent ion channels. It is well known that Ca^2+^ is involved in many important cellular response mechanisms as a major intracellular messenger [27,28,29]. The changes in intracellular Ca^2+^ concentration are closely related to the physiological and pathological mechanisms in important tissue systems; for example, alteration in calcium concentration in culture medium affects epidermal cell proliferation and differentiation [30].

It is widely acknowledged that the dynamic stability of extracellular and intracellular Ca^2+^ is beneficial to bone health and in maintaining the endocrine balance [31,32,33]. In recent years, intracellular Ca^2+^ overload has been regarded as key in a series of cardiovascular risks such as coronary artery diseases, arrhythmia, and cardiac failure; and clinically, calcium antagonists have been used effectively in treating angina, hypertension, and supraventricular tachyarrhythmias, which illustrates that maintenance of Ca^2+^ homeostasis confers cardioprotective benefits [34,35,36,37,38,39,40]. A majority of TRP channels are permeable to Ca^2+^ and play a unique role as cell sensors [41]. They are involved in various cellular functions mediated by Ca^2+^, such as contraction, proliferation, and apoptosis [42,43,44]. In other words, TRP channels act as gatekeepers in homeostasis [14,45,46,47,48]; for example, it has been reported that the TRP channels maintain the dynamic equilibrium between Mg^2+^ and Ca^2+^ in epithelial tissues [49].

The multiple functions played by the TRP channels and their activated multimodality characteristics imply that correct channel gating or infiltration may facilitate the study of complex pathophysiological mechanisms [1,14,16,50]. In the last ten years, several studies from Nilius et al., Watanabe et al., and Kaneko et al. have successively shown that TRP channels are highly expressed in the gastrointestinal tract, genitourinary system, immune system, endocrine system, respiratory system, nervous system, and cardiovascular system, which are involved in complicated physiological and pathological processes [12,13,14].

In contrast to other TRP family members, TRPM4 can be activated by an intracellular accumulation of Ca^2+^ [8,51]. In addition, TRPM4 is permeable to the monovalent cations with the following ionic selectivity: Na^+^ > K^+^ > Cs^+^ > Li^+^ [52]. However, TRPM4 shows no Ca^2+^ permeability, which induces intracellular Ca^2+^ accumulation and overload to cause depolarization of the cell membrane, which further leads to cell damage or death [2,15]. Therefore, we will systematically analyze the physiological and pathological role of TRPM4 in the cardiovascular system as far as the specificity of TRPM4 for the non-permeability of Ca^2+^ is concerned.

### 2.2. Comparison between TRPM4 and TRPM5

Additionally, TRPM is a multifunctional group of TRP channels. As well as other TRPs, the majority of TRPM members are cation channels with Ca^2+^-permeability, except for TRPM4 and TRPM5. It has been described that there is 50% amino acid sequence homology between the two channels [1,4]. The activity of TRPM5 is also initiated by a rise in the intracellular Ca^2+^, which is quite similar to TRPM4, also being involved in numerous physiological and pathological mechanisms by modulating Ca^2+^ homeostasis. For example, according to Banik et al., TRPM4 and TRPM5 are both essential in transduction of taste stimuli [53]. Additionally, TRPM4 and TRPM5 are regulated by voltage and PIP2. TRPM5 has been regarded as a heat-activated channel, which shows temperature sensitivity in the range of 15–35 °C. As a close homolog of TRPM5, the temperature sensitivity of TRPM4 may be similar to that of TRPM5; studies have shown that its voltage-dependent activation curve may turn toward a more negative potential with an increasing temperature [9,54,55,56]. 

In contrast to the similarities described above, there are several differences between TRPM4 and TRPM5. For example, the tissue distributions of TRPM4 and TRPM5 are widely divergent. In research by Fonfria and colleagues, compared to TRPM4 expression, TRPM5 expression was observed only in a limited number of tissues including the intestine, pancreas, prostate, kidney, and pituitary. On the other hand, TRPM4 is highly expressed in the heart, lung, brain, bone, and stomach [57]. TRPM4 is involved in action potential generation in mouse atrial cardiomyocytes [58]. In 2014, Demir et al. found that the TRPM4 gene expression was increased slightly after exposure to myocardial ischemia-reperfusion. In contrast, TRPM5 was not detected in the heart [59].

### 2.3. TRPM4 Structure

As a member of the TRP channel family, TRPM4 contains various transmembrane and cytosolic domains that form a three-decker structure [60,61]. There is a wide selectivity filter of TRPM4 that is permeable to the monovalent cations owing to the pore-forming area between the transmembrane domains S5 and S6 [4,52,62]. As reported previously, the N-terminal nucleotide-binding domain and the C-terminal coiled coil in the NH_2_ and COOH terminal regions influence the tetrameric composition of TRPM4, and two ATP-binding cassette transporter-like motifs at the N-terminal nucleotide-binding domain are involved in the inhibition of TRPM4 activity [60].

Moreover, there are several PKC phosphorylation sites, five CaM-binding sites, four Walker B motifs (which are putative ATP-binding sites), a putative PIP_2_-binding site, and a coiled-coil domain, all of which participate in the modulation of TRPM4 function [1,10,60].

### 2.4. Activation of TRPM4

As a Ca^2+^- and voltage-activated channel, there are diverse ways in which TRPM4 is activated, and we will discuss the mechanisms underlying TRPM4 activation in terms of the following modulators: PIP_2_, ATP, PKC-dependent phosphorylation, and CaM.

#### 2.4.1. PIP_2_

PIP_2_ is a minor phospholipid component of the cell membrane, where it is a substrate for a number of important signaling proteins [63,64]. As a substrate of phospholipase C (PLC), PIP_2_ regulates all types of ion channels and transporters, including the voltage-gated K^+^ and Ca^2+^ channels [65,66]. In 2005–2006, the studies of Zhang et al. and Nilius et al. showed that PIP_2_ is a powerful enhancer of TRPM4, which may lead to a desensitization effect on TRPM4 activity through PLC-mediated PIP_2_ decomposition [6,7]. With the hydrolysis of PIP_2_ in the plasma membrane due to Ca^2+^-mediated PLC activation, TRPM4 gradually becomes insensitive to Ca^2+^, which leads to a move toward the state of negative potential [6,9]. Under a Ca^2+^-desensitized condition, TRPM4 shows voltage-dependent gating with increased open-state probability at a depolarizing membrane potential [51,60]. It has been demonstrated that poly-l-lysine, which is a type of PIP_2_ scavenger, can trigger a sharp desensitization [7].

Previous studies have additionally shown that loss of PIP_2_ results in a rapid attenuation of the TRPM4 current, in accordance with Nilius et al., which has been detected by adding PIP_2_ or inhibiting PIP_2_ extracellularly of HEK293 cells, both of which can reverse the depletion of the TRPM4 current [6]. Furthermore, depression of PLC activity with U73122, an inhibitor of PLC [67], is also able to preserve the level of PIP_2_ [6,9,68].

In summary, PIP_2_ is a critical auxiliary factor in the activation of TRPM4. PIP_2_ cannot activate TRPM4 on its own, but can rectify desensitization, increase the sensitivity of TRPM4 to Ca^2+^, and restrict the voltage dependence of TRPM4 [6,7,10,69,70]. To date, two PIP_2_-binding sites have been discovered at the C-terminal of TRPM4, which implies that the alteration of these binding sites may affect the sensitivity of TRPM4 to PIP_2_ and Ca^2+^ [6,71].

#### 2.4.2. ATP

Owing to the structure of TRPM4, Ca^2+^ sensitivity of TRPM4 can be modulated by ATP, PKC phosphorylation, and binding of CaM to the C-terminus [8,9,10,11]. ATP can restore the Ca^2+^ sensitivity of TRPM4 after desensitization has been clarified previously [8]. In the absence of Ca^2+^, TRPM4 can revert from desensitization when the cytoplasmic side of the membrane is exposed to Mg^2+^-chelated ATP [8,9]. However, TRPM4 currents would attenuate in the case of ATP deficiency after recovery. Therefore, addition or depletion of ATP has become vital in regulating the activation of TRPM4. As mentioned earlier, the ATP-binding sites, which include two Walker B motifs in the N-terminus and ABC transporter signature motifs, can be predicted from the amino acid sequence of TRPM4 [8,72,73]. It was also reported that TRPM4 currents are eliminated sharply when all the sites predicted to affect the ATP-binding of the channel carry mutations, which suggests that ATP is involved in the preservation of TRPM4’s Ca^2+^ sensitivity [8].

#### 2.4.3. PKC Phosphorylation

PKC-mediated TRPM4 phosphorylation can increase the Ca^2+^ sensitivity of TRPM4. A PKC activator, phorbol 12-myristate 13-acetate (PMA), can reduce the Half maximal effective concentration (EC_50_) level of Ca^2+^ in TRPM4 from 15 to 4 μM [8,74], which suggests that the increased activity of PKC helps improve the Ca^2+^ sensitivity of TRPM4 and resist its desensitization. This effect of PMA disappears when both the readily-phosphorylated amino acid residues, S1145A and S1152A, at the C-terminus of TRPM4 carry mutations [8].

#### 2.4.4. CaM 

A previous study by Nilius et al. found that dominant-negative mutants of CaM reduce the activation of TRPM4. CaM partially counters the reduction of Ca^2+^ sensitivity of endogenous TRPM4. Mutations in the C-terminal binding site of CaM can significantly reduce TRPM4 current amplitude and promote faster current decay. This suggests that the C-terminal binding site of CaM is vital for the Ca^2+^ sensitivity of TRPM4 [8,75].

The following sections discuss why it is important to understand TRPM4’s role in diseases by exploring the activated mechanism of the channel.

### 2.5. Inhibitors of TRPM4

Flufenamic acid is one of the inhibitors of TRPM4 and widely used in research [1]. Originally, according to Winder et al., flufenamic acid was identified in the 1960s as a member of the anthranilic acid-derivative class of nonsteroidal anti-inflammatory drugs (NSAID) due to its anti-inflammatory property, which is able to inhibit the production of prostaglandins. Subsequently, it has been found that flufenamic acid could act as an ion channel regulator that mainly impacts the nonselective cation channels. The TRPM4 channel can be significantly inhibited by flufenamic acid in the range of 4–12 μM. However, it affects sodium, potassium, calcium, and chloride channels as well [76].

Glibenclamide is another TRPM4 inhibitor, which can block TRPM4-like currents in sinoatrial node cells completely at a concentration of 100 μM [77]. The inhibiting effect of glibenclamide on TRPM4 channel is weaker than that on ATP-dependent K^+^ channels, which are vital therapeutic targets of type II diabetes [78]. Compared to the chemicals above, 9-phenanthrol, which is a tricyclic aromatic compound, inhibits TRPM4 activity specifically [79,80].

## 3. TRPM4 and Cardiovascular Disease

### 3.1. TRPM4 and Arrhythmia

Arrhythmia is a group of conditions with abnormal frequency of cardiac pulsation and/or rhythm, caused in the origin of heart activity and/or conduction disturbances, such as atrial sinus node dysregulation, excitement outside the sinoatrial node, agitation, slow conduction, and blocked or abnormal channel conduction [81,82,83,84]. Arrhythmia is an important class of diseases with respect to cardiovascular risk. The reverse transport of the sodium–calcium exchanger (NCX) on cardiomyocytes is one of the major pathways that leads to intracellular Ca^2+^ overload [85,86,87,88,89]. The research of Voigt et al. in 2011 demonstrated that increased NCX expression and activity can delay afterdepolarization, which triggers and promotes chronic atrial fibrillation [86,90,91,92]. Interestingly, the coupling of TRP channels with NCX proteins (TRP–NCX-mediated Ca^2+^ signaling) is able to disrupt intracellular Na^+^ and Ca^2+^ concentration, which produces a sudden accumulation of intracellular Ca^2+^, especially in the case of TRPC3 [93,94,95].

It is clear that TRPM4 is highly expressed in atrial cardiomyocytes [1,77]. Being a member of the TRP channel family, it is worth considering whether a coupling between TRPM4 and reverse mode-NCX may induce atrial arrhythmias [12]. In 2009, a *TRPM4* mutation was detected in a patient with hereditary heart disease: the glutamate at position 7 was replaced with lysine, which induced progressive cardiac bundle-branch block [96]. This is the first case to closely link *TRPM4* mutation to the pathophysiology of human cardiovascular disease [1,96]. The researchers subsequently demonstrated that although these mutations did not change the physiological characteristics of TRPM4, the SUMOylation—a post-translational modification to regulate the protein function by combining a member of the small ubiquitin-like modifier (SUMO) family and the target protein—of the mutant protein caused the alternation of TRPM4 protein expression and its current level at the plasma membrane [16,17,18,97].

However, the link between the function of TRPM4 and cardiac conduction block remains unclear. Perhaps the dysfunction of the voltage-dependent Na^+^ channel takes place, considering that TRPM4 switches on the depolarization of the cell membrane potential, which subsequently injures the cardiac conduction system [52,98,99]. Recently, other studies have also illustrated that *TRPM4* mutations are associated with isolated cardiac conduction disease, right bundle-branch block, tachycardia, and Brugada syndrome [17,18,19,20,21].

In summary, the fluctuation of Na^+^ and Ca^2+^ is involved in the regulation of the cardiac conduction system and is closely linked to the development of arrhythmia. As a result, TRPM4, which is an important Ca^2+^ regulator, may also be involved in cardiac conduction system diseases.

### 3.2. TRPM4 and Cardiac Hypertrophy

Cardiac hypertrophy is one of powerful adaptive mechanisms of the cardiovascular system, which is mainly manifested by an increase in cell volume and weight [100,101,102]. Adaptive alteration, which is so-called physiological hypertrophy, occurs to meet the increased demand of blood circulation. Under the physiological condition, the cardiac function can be performed in an orderly manner [93]. However, if it exceeds the range of compensation owing to cardiac pressure or volume overload, such as high levels of angiotensin II (AII), chronic hypertension, valvular dysfunction, myocardial infarction, and even excessive training, the hypertrophy shifts to an irreversible pathology, which induces severe arrhythmia or cardiac failure [22,103,104,105,106].

Calcium is indispensable in the excitation–contraction coupling and heart contractility under normal physiological conditions [87,88]. In the opinion of Lopea et al. in 1997, intracellular Ca^2+^ levels in the cardiomyocytes of humans with myocardial hypertrophy were sustained at high levels [107], which might be due to the following two methods: First, the activation of the phosphoinositide-specific PLC pathway leads to the generation of inositol 1,4,5-trisphosphate and diacylglycerol (DAG) via the hydrolysis of PIP_2_, both of which function as secondary messengers mediating the release of Ca^2+^ from the smooth endoplasmic reticulum and sarcoplasmic reticulum [22,108,109,110]. Second, the nuclear factor of activated T cells (NFAT), one of the transcription factors regulated by calcium signaling, also takes part in the regulation of decompensated cardiac hypertrophy. CaM activates serine/threonine phosphatase calcineurin (CN), and activated CN rapidly dephosphorylates the serine-rich region and serine-proline (SP)-repeats in the amino termini of NFAT, resulting in its translocation from the cytoplasm to the nucleus and producing pathological cardiac hypertrophy [111,112,113,114]. There is no doubt that Ca^2+^ plays a significant role in inducing cardiac hypertrophy. Therefore, if the mechanisms that maintain intracellular Ca^2+^ homeostasis are perturbed, the evolution of the hypertrophic alteration at the stage of compensation and decompensation in the myocardium is affected. Thus, calcium-regulated ion channels act as typical mediators.

TRP channels are important targets in myocardial remodeling because they are capable of transmitting long-term calcium signals [115,116,117]. Studies have shown that TRPM4 seems to be involved in the occurrence and development of cardiac hypertrophy, in accordance with Demion et al., Kecskés et al., and Guefer et al. [104,118,119]. Indeed, TRPM4 has been shown to play a role in cardiac hypertrophy by the construction of a typical model that mimics the ventricular hypertrophic alteration in spontaneously hypertensive rats (SHRs) [120]. Changes in TRPM4 current and mRNA levels are detectable in SHRs. Compared with WKY rats (control group), the expression levels of TRPM4 mRNA in SHRs were almost 50-fold.

Although a significant level of TRPM4 current exists in SHRs, it is quite challenging to find it in WKY rats even by facilitating the TRPM4 current with application of PKC. TRPM4 properties can be monitored by the Patch-Clamp measurement to illustrate the existence of a nonselective cation channel in ventricular cardiomyocytes isolated from SHRs. Thus, TRPM4 is crucial during the process of ventricular remodeling [22,120].

TRPM4 is a negative regulator of cardiac hypertrophy induced by angiotensin II (AII) stimulation [104]. In this previous study, the researchers selectively removed the exons 15 and 16 from *TRPM4* for establishing a cardiac-specific TRPM4-knockout mouse line (TRPM4^cKO^). They intended to compare cardiac hypertrophic changes between TRPM4^cKO^ mice and wild-type mice (littermate controls; TRPM4^+/+^) under AII stimulation. Their results showed significantly higher cardiac hypertrophy at the morphological level in TRPM4^cKO^ mice compared with the wild-type mice, including increased heart size and weight. In addition, high levels of hypertrophy-related genes such as atrial natriuretic peptide (*ANP*), *α-actin*, and *Rcan1* were detected in the TRPM4^cKO^ mice.

TRPM4 deficiency increases the hypertrophic response to chronic AII in cultured neonatal myocytes. Increased intracellular Ca^2+^ concentration in isolated ventricular cardiomyocytes of TRPM4^cKO^ mice after AII stimulation reconfirmed that TRPM4 is a key regulator of intracellular Ca^2+^. There was also a significant rise in CN phosphatase activity in the hearts of TRPM4^cKO^ mice following AII treatment. In these mice, Rcan1 mRNA, which is a reporter of the calcineurin–NFAT activation typical in cardiac hypertrophy, is overexpressed. Additionally, store-operated calcium entry (SOCE), which is helpful in promoting intracellular Ca^2+^ collection from the extracellular environment, has been regarded as having an important role in mediating cardiac hypertrophy [104,117,121]. The study of Kecskés et al. showed that AngII-mediated SOCE was increased in Trpm4^−/−^ myocytes [104]. It means that TRPM4 is related to modulation of the Ca^2+^ influx through SOCE to induce pathological hypertrophy [104,117,121] 

TRPM4 is important for the beneficial cardiac remodeling induced by endurance training, and the role of TRPM4 in physiological and pathological cardiac hypertrophy has also been studied [118]. In TRPM4^+/+^ mice, enhanced cardiac function in response to endurance training was observed. In contrast, a high number of apoptotic DNA fragments are detectable in TRPM4^−/−^ mice after endurance training. This illustrates that TRPM4 can obstruct the progress from adaptive hypertrophy to irreversible cardiac remodeling. In the pathological cardiac hypertrophy, Ca^2+^ entry via SOCs causes activation of the calcineurin–NFAT pathway. The TRPM4 channel inhibits the SOCs and thus prevents pathological remodeling [118].

The link between TRPM4 and arrhythmia, wherein 9-phenanthrol can inhibit TRPM4 and can shorten the duration of action potentials (APs), has been confirmed previously, which implies that TRPM4 can delay AP repolarization [119]. It has been hypothesized that TRPM4 is implicated in the prolongation of AP duration, which may be present in the hypertrophied heart, but has not been verified to date [122,123,124].

Thus, TRPM4 is an essential regulator of the hypertrophic alteration and is likely to supply a novel direction for the treatment of cardiovascular diseases caused by cardiac hypertrophy [22,122].

### 3.3. TRPM4 and Myocardial IRI

Ischemic heart diseases (IHDs) have become the most frequently reported threat to human health [125,126]. Myocardial ischemia originates owing to a reduction in coronary blood flow, which may result in an imbalance between myocardial supply and demand of oxygen. Typical symptoms include chest pain, chest discomfort, weakness or dyspnea after exercise, and mental stimulation or overeating, which are detrimental to the quality of life. Restoring the blood flow is an effective therapeutic strategy to treat ischemia, as achieved through thrombolysis, coronary artery bypass graft, and percutaneous coronary intervention. However, secondary injuries after recanalization are risk factors to IHD patients, such as contractile dysfunction, arrhythmia, and sudden death, which are also known as myocardial IRI [127,128,129].

The mechanism underlying myocardial ischemia-reperfusion injury (IRI) is complex [130,131,132]. At the onset of ischemia, ATP decreases progressively, which subsequently causes a dysfunction of ion pumps, leading to the accumulation of intracellular Ca^2+^, especially in the mitochondria. This promotes the depletion of ATP with increased Ca^2+^, leading to cell death. With the return of blood flow, even if it is conducive to the recovery of ATP, a large burst of Ca^2+^ and reactive oxygen species in the mitochondria occurs at the onset of reperfusion, which opens the mitochondrial permeability transition pore and causes further myocardial damage. Thus, reduced ATP and disrupted calcium metabolism eventually lead to myocardial IRI. It has also been identified that Ca^2+^ increase and ATP depletion are involved in the activation of TRPM4, and a link between TRPM4 and myocardial IRI has been reported [36,130,131,132,133,134].

Inhibition of TRPM4 with 9-phenanthrol (9-Phe) has been shown to confer cardioprotective effects against IRI in the rat heart [24]. In the Langendorff-perfused rat heart, the TRPM4 inhibitor 9-Phe produced a dramatic recovery of the damaged left ventricular contractile function caused by experimental IRI. Furthermore, 9-Phe treatment could effectively resist the ventricular fibrillation induced by IRI. This showed that damaged cardiac contractile function caused by IRI could be rescued through the inhibition of TRPM4, which may confer cardioprotection mainly via an anti- arrhythmic effect. After IRI, the infarcted area of myocardium was smaller in the rats treated with 9-Phe, which further elaborated that regulation of TRPM4 is important in reversing the myocardial damage induced by IRI [24].

The relationship between TRPM4 and myocardial IRI has been explored more deeply in vitro as well [23]. In rat ventricular cardiomyocytes H9c2, 9-Phe treatment prevented cellular damage caused by two types of IRI models: one being reactive oxygen species stimulation (ROS) by hydrogen peroxide (H_2_O_2_) exposure, and the other being hypoxia/reoxygenation (H/R) induction. Moreover, the viability of cardiomyocytes with the TRPM4 knocked down through small interfering RNA (siRNA) transfection was significantly higher than that of the nontransfection group following ROS damage and H/R induction. It can also be suggested that inhibition of TRPM4 protects cardiomyocytes against IRI both in vivo and in vitro, and 9-Phe treatment confers remarkable cardioprotection against oxidative stress injury. Therefore, inhibiting TRPM4 might be promising in treating myocardial IRI for IHD patients [23].

TRPM4 is closely related to intracellular Ca^2+^ dynamic equilibrium, and the mechanism of intracellular Ca^2+^ overload-induced IRI has already been elucidated [8,116,122,135]. Therefore, the role of inhibiting TRPM4 in ameliorating an abrupt increase in intracellular Ca^2+^ during myocardial IRI is being explored.

### 3.4. TRPM4 and Endothelial Cell Injury and Apoptosis

Endothelial injury is the risk factor and may cause a series of cardiovascular system disorders [136,137]. Due to the research conducted by Gerzanich et al., Becerra et al., and Ding et al., the expression of TRPM4 could be upregulated when the vascular endothelium is damaged under a variety of pathological conditions. For example, TRPM4 expression levels increase by more than 33% in endothelial cells damaged owing to a high-salt diet [138,139,140,141]. Moreover, the expression of TRPM4 in both protein and mRNA forms increases in human umbilical vein endothelial cells under hypoxic and ischemic conditions [10,142]. It has also been shown that TRPM4 is involved in lipopolysaccharide-induced vascular endothelial injury [139]. Thus, inhibiting the expression or activity of TRPM4 via 9-Phe and glibenclamide can effectively protect vascular endothelial cells against lipopolysaccharide-induced damage.

In a lipopolysaccharide-treated group, more than 40% of the endothelial cells were apoptotic, whereas the apoptosis in the TRPM4-inhihition group reduced significantly [139]. Lipopolysaccharide induces endothelial cell death because it promotes a rise in intracellular ROS [143]. Furthermore, the expression of TRPM4 is significantly upregulated upon treatment with exogenous ROS (H_2_O_2_), which results in endothelial cell apoptosis. However, this could be reversed through the inhibition of TRPM4 activity with 9-Phe [138]. Thus, it is implied that the involvement of TRPM4 in endothelial injury is also mediated by ROS [131,133,134,144].

It is clear that under the influence of various vascular injury factors, the upregulation of TRPM4 in vascular endothelial cells produces complete or maximal depolarization, resulting in the continuous influx of sodium ions [137,138,139]. Soon afterwards, the rise of intracellular Na^+^ and Cl^−^ is able to cause enlargement of osmotic pressure, which drives the influx of H_2_O, inducing cell swelling or rupture [140].

## 4. Conclusions

Overall, TRPM4 is an important nonselective cation channel involved in cellular calcium regulation. A majority of cardiovascular diseases are closely related to calcium homeostasis. As the research on TRPM4 progresses, more functions will be recognized and intrinsic links between TRPM4 and the development of cardiovascular diseases may be discovered.
